# The Role of Antifungals in Pediatric Critical Care Invasive Fungal Infections

**DOI:** 10.1155/2018/8469585

**Published:** 2018-11-22

**Authors:** Ashlesha Kaushik, Helen Kest

**Affiliations:** ^1^Pediatric Infectious Diseases, Unity Point Health and Siouxland Medical Education Foundation, Department of Pediatrics, University of Iowa Carver College of Medicine, 2720 Stone Park Blvd, Sioux City, IA 51104, USA; ^2^Pediatric Infectious Diseases, Department of Pediatrics, St. Joseph's Children's Hospital, 703, Main Street, Paterson, NJ 07503, USA

## Abstract

Invasive fungal infections (IFIs) have seen considerable increase in pediatric intensive care units over the past several decades. IFIs are predominantly caused by *Candida* species, and candidemia is the third most common cause of healthcare-associated bloodstream infections (BSIs) in children. IFIs are opportunistic infections that affect pediatric patients in critical care resulting in significant morbidity and mortality especially in those with a compromised immune system. IFIs are the leading cause of death in children with comorbidities such as immunosuppression, and pediatric ICU admission has been shown to be an independent risk factor for mortality. Management of IFI and fungal sepsis is broad and encompasses several key components that include prompt initiation of therapy and rapid source identification and control. This study reviews important antifungals in the pediatric critical care setting including the pharmacologic properties, antifungal spectrum, adverse effects, and clinical uses of agents belonging to the four major classes of antifungals—the polyenes, azoles, echinocandins, and pyrimidine analogue flucytosine. The polyenes and azoles are the most often used classes of antifungals. The echinocandins are a relatively newer class of antifungal agents that offer excellent *Candida* activity and are currently recommended as the first-line therapy for invasive candidiasis.

## 1. Introduction

Fungi are ubiquitous organisms that rarely cause disease in otherwise healthy immunocompetent hosts. Of the millions of different fungal species, only few (about 300) are known to cause disease [1]. IFIs result from the interplay between the organism's pathogenic ability at colonization, adaptation, propagation, and/or dissemination and the host's immune defense and response. Therefore, effective management of IFI will include efforts aimed at correcting underlying defects [2]. IFIs are predominantly caused by *Candida* species. Candidemia is the third most common cause of healthcare-associated bloodstream infections in children [3].

IFI has increased especially in the critical care setting in the last two decades as a result of greater use of broad-spectrum antibiotics and rise in the use of invasive procedures [3, 4]. IFIs are the leading cause of death in children with comorbidities and immunosuppression, and pediatric ICU admission has been shown to be an independent risk factor for mortality [5].

The three major pathogenic factors for the development of an IFI include compromised natural barriers resulting from mucositis, invasive procedures and indwelling catheters, defects in cell-mediated immunity related to T-cell cytotoxic agents, and myelosuppression and decreased phagocytes as a result of chemotherapy [6, 7]. High-risk groups for developing IFIs include patients undergoing hematopoietic stem cell transplantation (HCT) especially with an allogeneic donor; patients receiving chemotherapy for acute myeloid leukemia (AML) or relapsed acute lymphoblastic leukemia (ALL); and patients with severe aplastic anemia [8].

In pediatric oncology patients, with the availability of better chemotherapy protocols aiming at increased survival, there is a resultant risk of increase in serious and disseminated fungal infections even with ubiquitous and normally innocuous fungal pathogens. The intensive immunosuppressive regimens cause disruption of normal host defenses including mucosal barriers, thus predisposing to IFIs.

Rheumatologic and connective tissue disorders needing immunosuppressive therapy, congenital immunodeficiency syndromes, and acquired immunodeficiency states like HIV/AIDS are other predisposing conditions for developing IFIs.

Pediatric IFIs pose a significant economic burden to the U.S. health-care system with a mean increased inpatient stay of ≥ 21 days and about $92,000 in excess hospital costs [9].

Additionally, disease recognition of IFI in pediatrics poses another huge challenge due to the nonspecific clinical signs and symptoms. Fever without a focus is the most common presentation. In high-risk groups, a high index of suspicion for IFI is required for prompt initiation of targeted antifungal therapy. This has led to development of protocols and guidelines that are easily accessible to the critical care practitioner for fungal prophylaxis, empiric and preemptive therapy [7, 9, 10]. Histopathologic examination (with special stains) demonstrating fungal tissue or isolation from sterile clinical specimens remains the main stay of diagnosis. Deep-seated infections typically require surgical debridement with systemic therapy, and other adjunctive treatments like immunotherapy may be indicated.

While still not widespread, recent trends show that IFI caused by multidrug-resistant *Candida* and *Aspergillus* species are increasingly becoming a global public health threat. Use of antifungals (prophylactic, preemptive, empiric, and culture directed) in the critically ill patient population has led to improved outcomes and survival [11, 12].

## 2. Antifungal Drug Classes

Once the decision to begin therapy is made, the next step in management is choosing the optimal regimen and dosing. This decision is driven by disease-based risk stratification, mechanism of action, spectrum of activity, pharmacological properties, and adverse effects/toxicities. Currently, there are four classes of antifungals that are approved by the U.S. Food and Drug Administration (FDA) for IFI.  Table 1 shows the US FDA approval timeline of various antifungal agents [13–15].

These include the polyene group that encompasses amphotericin B deoxycholate and its lipid formulations (liposomal amphotericin B, amphotericin B lipid complex, and amphotericin B cholesteryl sulfate complex); the nucleoside analogue flucytosine; the triazoles which include fluconazole and voriconazole; and the most recently developed echinocandin class, which includes caspofungin, micafungin, and anidulafungin.

Amphotericin B deoxycholate was the first antifungal approved in 1958. However, significant renal toxicity with transfusion reactions led to a more limited and cautious use [14]. 5-Flucytosine, active against *Candida* species and *Cryptococcus neoformans*, was approved in 1972, but its use became problematic due to high incidence of resistance and toxicity. First-generation azole drugs followed with better toxicity profiles, advantageous oral routes, and good antifungal activity. Their limitations included potential for multiple drug-drug interactions due to CYP450 interactions. Lipid-based amphotericin B formulations were introduced in the 1990s with better toxicity profiles that led to expansion of amphotericin use. Echinocandins are the newer antifungals offering excellent *Candida* activity and are currently recommended as the first-line therapy for IFI while awaiting final determination and/or cultures. Second-generation azole drugs, including voriconazole, posaconazole, and isavuconazole, show improved extended spectrum of activity against filamentous fungi.

Table 2 shows the spectrum of activity of antifungals against important fungi causing invasive disease [13, 14].

## 3. Susceptibility Testing

Despite its still less than optimal availability, use of antifungal susceptibility testing (AFST) in critical care constitutes an indispensable though challenging tool in IFI management. In the ever challenging world of managing IFI, determination of MICs is a proxy for antifungal agent efficacy and provides susceptibility information for local epidemiologic data and effective empiric antifungal choices.

Drawbacks include limitations of common referenced AFST methods like Clinical Laboratory Standards Institute (CLSI) or European Committee on Antimicrobial Susceptibility Testing (EUCAST) with its available commercial adaptations due to slow turnaround time. Additionally differing clinical breakpoints and their subjective interpretations, issues with correlation with efficacy and inter-reader subjectivity in visual MIC determination may lead to unreliable results in the clinical setting [16].

Newer nucleic acid-based technologies are becoming increasingly used for the detection of antifungal resistance and newer generation methods like genomic analysis may become a critical tool for predicting evolving population based epidemiologic patterns as well as tailored susceptibility and level of resistance [17, 18].

## 4. Systemic Antifungal Agents in Invasive Fungal Infections That Act on Fungal Cell Membranes: Polyenes and Azoles

### 4.1. Polyenes

The polyenes are the oldest antifungals and are natural products of a soil actinomycete, *Streptomyces nodosus* [15]. This class consists of a single agent, amphotericin B. Multiple lipid-based formulations have been designed and developed to limit its toxicity.

Polyenes are lipophilic compounds acting on the cell membrane and are fungicidal in action. Amphotericin B binds to ergosterol, the major sterol in the fungal cell membrane, creating transmembrane channels with resultant increase in permeability of monovalent cations and cell death by osmotic lysis [13–15].

### 4.1.1. Amphotericin B Deoxycholate

Amphotericin B deoxycholate is also called “conventional” amphotericin B.

*(1) Pharmacokinetics*. Amphotericin B attains high concentrations in tissues like liver, spleen, and lungs. However, cerebrospinal fluid (CSF) concentrations are low (2–4% of serum concentrations). The antifungal's initial 24–48 hour half-life reflects uptake by lipids, a slow release, and excretion into urine and bile. The drug then undergoes metabolism with a terminal half-life of fifteen days. Amphotericin B (AmB) demonstrates concentration-dependent killing with a prolonged postantifungal effect. Therefore, large daily doses are the most effective, and achieving optimal peak concentrations is an important consideration. Moreover, there is progressive accumulation of the drug with continued administration as suggested by the relationship between the total dose administered and tissue concentrations.

There is no evidence-based support for using higher doses of AmB >1.5 mg/kg/d, and higher doses are associated with greater toxicity without improving efficacy.

*(2) Adverse Effects of Amphotericin B*. AmB interacts with cholesterol in human cell membranes accounting for the adverse effects and toxicities. Adverse effects of AmB include fever, nephrotoxicity, hypokalemia, magnesium wasting, and anaphylaxis [13, 19].

A large proportion of patients receiving AmB experience infusion-related reactions or renal toxicity. Nephrotoxicity is especially common when AmB is given along with other nephrotoxic drugs like loop diuretics. However, AmB-related nephrotoxicity is usually reversible with renal function returning to normal after discontinuation of the drug.

### 4.1.2. Amphotericin B Lipid Formulations

Several lipid formulations are available like

1. AmB lipid complex (ABLC) (Abelcet®)
2. Liposomal AmB (L-AmB) (Ambisome®)
3. AmB colloidal dispersion (ABCD) (Amphocil®/Amphotec®): no longer available in the U.S. due to toxicity

Lipid formulations were developed with the objective of decreasing the adverse effects of conventional AmB. Essentially in lipid formulations, AmB is not available to interact with the cholesterol of cell membranes of humans as the complex with lipids stabilizes AmB in a self-associated state [14].

The reduced renal toxicity of lipid formulations is likely attributed to the preferential binding of its amphotericin to serum high-density lipoproteins as opposed to binding of conventional AmB to low density lipoproteins in serum [14]. Liposomal AmB has less infusion-related toxicity than ABLC, while ABCD has dose-limiting infusion-related reactions and appears closer in toxicity to conventional AmB [20]. The main advantages of amphotericin B lipid formulations over conventional AmB include increased daily dose of parent drug (3–5 mg/kg/day), slower onset than conventional AmB in time-kill studies, equivalent efficacy to conventional AmB in treatment of IFI, improved distribution in reticuloendothelial organs (lungs, liver, and spleen), and reduced toxicity.

*(1) Indications for Amphotericin B as the Drug of Choice*. Amphotericin B is the preferred drug for disseminated/severe fungal diseases including cryptococcosis, coccidioidomycosis, paracoccidioidomycosis, histoplasmosis, blastomycosis, mucormycosis (zygomycosis) for induction therapy, and sporotrichosis [4, 5]. However, it should not be used as the primary drug for aspergillosis. Major uses in the critical care setting and pediatric dosage of AmB as well as of lipid formulations are summarized in Table 3 [13, 21].

*(2) Indications for In Vitro Antifungal Testing of Amphotericin B*. The indications for susceptibility testing are rare and include clinical failure or infection with pathogens known to be resistant to AmB like *Candida lusitaniae*, *Trichosporon* spp., *Fusarium* spp., *Pseudallescheria boydii* (asexual form *Scedosporium apiospermum/Scedosporium prolificans*).

### 4.2. Azoles

This class can be subdivided into the older generation imidazoles and the newer generation triazoles, based on the number of nitrogen atoms in the azole ring. The structural differences lead to different affinities for the cytochrome P450 enzyme system.

#### 4.2.1. Imidazoles

- Miconazole (*superficial use only*)
- Clotrimazole (*superficial use only*)
- Ketoconazole

#### 4.2.2. Triazoles

- Fluconazole
- Itraconazole
- Voriconazole
- Posaconazole
- Isavuconazole
- Ravuconazole
- Albaconazole

#### 4.2.3. Mechanism of Action

Ergosterol is responsible for integrity of the fungal cell membrane based on its bioregulatory activity on membrane fluidity and asymmetry [19]. Azoles inhibit fungal cytochrome P450-dependent 14*α*-demethylation of lanosterol, leading to substitution of methylated sterols in the membrane and depletion of ergosterol, which results in an accumulation of precursors with abnormalities in the fungal membrane. Pharmacodynamic studies demonstrate time-dependent antifungal activity optimized at concentrations 1-2 times the MIC [11–13]. Additionally, there is prolonged ongoing growth suppression after triazole concentrations decrease to less than the MIC indicating prolonged postantifungal effect like the polyenes group. No increase is seen once the maximal fungistatic concentration is attained for azoles. Onset of activity of azoles is not as rapid as AmB since inhibition of sterol synthesis takes longer than directly creating channels. Resistance is conferred by genetic regulation of target enzyme and/or reduced access. Azoles are generally fungistatic agents but show fungicidal activity against *Aspergillus* species. Major uses of azoles (fluconazole and voriconazole) and dosages in the pediatric critical care setting are summarized in Table 4 [13, 22].

#### 4.2.4. Fluconazole

*(1) Pharmacokinetics*. Oral fluconazole is approximately 90% bioavailable and passes into tissues and fluids very rapidly, because of decreased lipophilicity and plasma protein binding. Fluconazole activity is not concentration dependent, and the drug achieves high concentrations in the CSF and vitreous humor (approximately 80% of those found in blood). Fluconazole is appropriate for treatment of fungal urinary tract infections as fluconazole concentrations in the urine are 10–20 fold higher than blood. Metabolism plays a minor role in fluconazole clearance, and the unchanged drug is predominantly cleared by the kidneys [14]. There is need for gastric acidity for absorption. Fluconazole may increase blood levels of various medications although to a lesser extent than with mould-active triazoles; however, there is no effect on testosterone or cortisol levels.

*(2) Indications*. Indications for fluconazole include candidiasis in nonneutropenic patients, esophageal candidiasis, maintenance therapy for cryptococcosis, mild or moderate coccidioidomycosis, sporotrichosis, and as a possible alternative for neurological disease in blastomycosis. It is notable that fluconazole has no mould activity. The major uses of fluconazole in the pediatric critical care setting are summarized in Table 4.

*(3) Indications for In Vitro Antifungal Testing of Fluconazole*. The indications include clinical failure and infections with fluconazole-resistant pathogens, including *Candida krusei* (inherent resistance) and *Candida glabrata* (dose-dependent).

#### 4.2.5. Itraconazole

Itraconazole is available as capsules, oral cyclodextrin suspension, and intravenous forms. Capsules are not recommended due to variable absorption, while the suspension is better absorbed. Elimination of cyclodextrin is dependent on glomerular filtration, so the vehicle (cyclodextrin) may accumulate in patients with significant impairment of renal function. Itraconazole levels need to be monitored while on therapy.

*(1) Pharmacokinetics*. Itraconazole demonstrates a high volume of distribution and accumulation in tissues. The primary route of elimination is hepatic, so there is no need for dose adjustment of itraconazole in renal failure. Patients with achlorhydria or those receiving H2-receptor antagonists may demonstrate impaired absorption. Administration of the capsule with acidic beverages (cola or cranberry juice) may lead to enhanced absorption. The absorption of the capsule formulation is significantly increased when administered with food; however, the oral suspension is better absorbed on an empty stomach [14].

*(2) Indications*. Itraconazole is the drug of choice for uncommon IFI like mild/moderate coccidioidomycosis, paracoccidioidomycosis, histoplasmosis, blastomycosis, sporotrichosis, and pseudallescheriasis. Some studies show that it may be used for mild aspergillosis disease.

*(3) Adverse Effects*. The most common adverse effects are gastrointestinal and include nausea/vomiting noted in 10% and elevated liver enzymes in 5% of patients [14]. Gastrointestinal side effects are more common with itraconazole than fluconazole. Since it is a potent inhibitor of the fungal CYP3A4 enzyme, it is advisable to avoid concomitant administration of rifampin, phenytoin, carbamazepine, and phenobarbital. Concomitant use with cyclophosphamide results in toxic cyclophosphamide metabolite generation and should be avoided.

#### 4.2.6. Voriconazole

Voriconazole is the newer generation triazole which is a fluconazole derivative, with the antifungal spectrum resembling itraconazole and pharmacokinetics similar to fluconazole. It is fungicidal with excellent mould activity and good CSF penetration. Voriconazole trough levels need to be monitored while on therapy. Oral formulations of voriconazole should be taken on an empty stomach, as food decreases levels. Voriconazole demonstrates a high variability in pharmacokinetics and levels between different patients.

Like fluconazole, voriconazole also has wide tissue distribution including the CSF and vitreal fluid; however, it is not excreted into urine and therefore is not effective for *Candida* cystitis.

*(1) Pharmacokinetics*. Voriconazole has a nonlinear pharmacokinetics in adults. The usual loading dose is 6 mg/kg/dose BID, and maintenance dose is 4 mg/kg/dose BID. Oral bioavailability is 96%. On the other hand, voriconazole pharmacokinetics is linear in children, with a loading dose of 9 mg/kg/dose BID (IV) and a maintenance dose of 8 mg/kg/dose BID (IV) or 9 mg/kg/dose BID orally. The bioavailability is about 50%. It is both a substrate for and inhibitor of human cytochrome P450 isozymes CYP2C19, CYP2C9, and CYP3A4.

While CYP2C19 family 2, subfamily C, polypeptide 19 (CYP2C19) accounts for about 5% of drug metabolism, its involvement in metabolism of agents in several therapeutic classes used for high-risk groups leads to therapeutic and toxicity concerns in clinical practice [23–25].

Wang et al. showed that pharmacokinetics of voriconazole differed across groups of healthy volunteers [24]. Total body clearance was six and two times higher in ultrarapid metabolizers and extensive metabolizers respectively [25].

Standardized phenotype dose adjustments are not in widespread use in the pediatric population; however, similar association between voriconazole plasma concentration and the CYP2C19 phenotype will likely change future trends [25]. Poor metabolizer trait appears uncommon in white/black population. Use of sirolimus is absolutely contraindicated with voriconazole. Side effects include reversible visual disturbances (15%), transaminitis (10–15%), photosensitization (1–5%), loss of color differentiation, hallucinations, and QTc prolongation with low serum magnesium levels.

*(2) Indications for Voriconazole*. Voriconazole is the preferred therapy for aspergillosis, scedosporiosis, fusariosis, and pseudallescheriasis and alternative therapy for candidiasis.

Voriconazole has no activity against mucormycosis (zygomycosis). Indications of voriconazole in pediatric critical care are shown in Table 4.

#### 4.2.7. Posaconazole

Posaconazole is available as an oral suspension, extended release tablet, and an intravenous formulation.

*(1) Pharmacokinetics*. Posaconazole oral suspension requires food for increased absorption (ideally a high fat meal); however, less is needed with tablet form. Posaconazole has saturable kinetics, and more than 800 mg/day cannot be absorbed.

*(2) Indications for Posaconazole*. Posaconazole is a potent mould-active antifungal agent. Indications include antifungal prophylaxis in malignant patients and graft versus host disease (GVHD) patients, treatment of mucormycosis (zygomycosis) and also as the maintenance therapy after AmB induction therapy for mucormycosis, and as an alternative for aspergillosis and candidiasis.

Isavuconazole is a new azole approved for aspergillosis and mucormycoses in adults in 2015. This is not approved for pediatric use.

Two newer generation azoles currently still in investigational phases are ravuconazole and albaconazole. These azoles were originally developed to expand antifungal spectrum of activity and improve tolerability. They show promising use as viable options for resistant IFI and other emerging fungal pathogens. However, cross-resistance with other azoles remains a concern. Albaconazole has excellent bioavailability and is available in oral forms; ravuconazole is available in both IV and oral forms [26–29].

Ravuconazole demonstrates high in vitro activity against major pathogenic fungi including fluconazole-resistant *Candida* species, *Cryptococcus neoformans*, *Aspergillus fumigatus*, and dermatophytes [26, 27].

Albaconazole shows activity against *Candida* species including *C. albicans*, *C. parapsilosis*, *C. tropicalis*, *C. glabrata*, *C. krusei*, and *C. guilliermondii* [28]. An added advantage is its additional activity against isolates of highly resistant difficult-to-treat *Scedosporium prolificans* [29]. Its favorable adverse effect profile includes absence of QTc prolongation that has been shown in other members of the azole class [29].

## 5. Antifungals That Inhibit Nucleic Acid Synthesis

### 5.1. Flucytosine (5-FC)

Flucytosine (5-FC) is the fluorinated analogue of cytosine. 5-FC gets converted into 5-fluorouracil (5-FU) within the fungal cells, and after incorporation into fungal RNA in place of uridylic acid, inhibits fungal protein synthesis. It also causes inhibition of thymidylate synthetase resulting in impaired fungal DNA synthesis.

Flucytosine has limited use in pediatric critical care and is only used in combination with other agents. It should never be used alone as antifungal resistance develops quickly to 5-FC monotherapy. Moreover, many fungi have intrinsic resistance to the drug.

#### 5.1.1. Clinical Applications of Flucytosine (5-FC)

5-FC distributes widely in tissues and facilitates antifungal activity of amphotericin B in sites with poor penetration for amphotericin B, including the CSF, cardiac valves, and vitreous humor. In turn, the penetration of 5-FC to the cell interior is facilitated by the membrane-permeability increasing effects of amphotericin B. Only oral formulation of 5-FC is widely available, and no intravenous formulation is available in the United States. 5-FC is recommended for cryptococcal meningitis with AmB [14]. 5-FC is no longer recommended for neonatal candidiasis.

#### 5.1.2. Flucytosine (5-FC) Toxicities

The toxicities affect mainly the bone marrow and gastrointestinal tract.

5-FC serum concentrations need to be closely monitored and trough levels must be maintained at approximately 40–80 *µ*g/ml. Since the drug is cleared renally, the interval between the doses need to be increased with renal dysfunction. 5-FC exacerbates myelosuppression in patients with neutropenia. Drug trough serum concentrations >100 *µ*g/ml are associated with bone marrow aplasia. Hematologic toxicity often appears in the first two weeks of initiating therapy.

## 6. Antifungals That Inhibit Cell Wall Synthesis

### 6.1. Echinocandins

This class is relatively novel and includes caspofungin, micafungin, and anidulafungin. All are generally equivalent in terms of efficacy, each with similar spectrum of activity.

They exhibit potent fungicidal activity against *Candida* species, including azole-resistant strains, and true clinical resistance to the echinocandins is rare. Combination regimens that include an echinocandin have shown promise in the treatment of aspergillosis. Major drawbacks are that echinocandins remain expensive to use and only parenteral formulations are available. Also, they are poorly distributed in the central nervous system, intraocular fluids, and urine.

#### 6.1.1. Mechanism of Action of Echinocandins

Echinocandins interfere with cell wall biosynthesis by noncompetitive inhibition of 1,3-glucan synthase, an enzyme present in fungi but absent in mammalian cells. This leads to decreased 1,3-glucan production, which is an essential cell wall polysaccharide that provides structural integrity to the fungal cell wall. They are fungicidal against *Candida*, but only create hyphal morphologic changes in *Aspergillus* and hence are fungistatic.

Additionally, contribution of biofilms to invasiveness of fungi has been well described in high-risk pediatric patients with devices or mucosal or other barrier disruption which contributes to the high burden of hospital-acquired infections [30]. Echinocandins show significant in vivo and in vitro activity against *Candida* biofilms compared to other antifungal agents like azoles and polyenes [31]. This has led to widespread use of echinocandins as first-line therapy for invasive *Candida* infections.

#### 6.1.2. Pharmacokinetics

Echinocandins are not metabolized through the CYP system, so they demonstrate less interactions and side effects than azoles. They do not accumulate in urine, hence are not effective for urinary tract infection, and there are no renal insufficiency dosing issues. A body surface area dosing is followed for caspofungin. Caspofungin follows triphasic kinetics, namely, tissue distribution that accounts for an initial rapid decline in plasma levels, followed by slow hepatic metabolism through hydrolysis and *N*-acetylation; it also undergoes spontaneous degradation; and it has a terminal half-life of 27 to 50 hours [14].

Micafungin has linear elimination kinetics and undergoes limited hepatic metabolism, with a half-life of 12 hours. For micafungin, clearance increases dramatically in younger age groups especially neonates, and the dose is 2–10 mg/kg/day. Anidulafungin does not undergo metabolism and is eliminated through spontaneous degradation with half-life of 40–50 hours [14].

Adverse reactions attributed to echinocandins are minimal because of lack of cross reactivity with human tissues since 1,3-*β*-glucan is specific to fungal cell walls and not found in human cells.

#### 6.1.3. Indications for Echinocandins

Clinical applications for echinocandins include candidiasis in patients with neutropenia, invasive candidiasis, candidiasis in severely ill patients, and neutropenic refractory esophageal candidiasis. Caspofungin is approved for empiric therapy of febrile neutropenia, and only micafungin is licensed for antifungal prophylaxis in stem cell transplantation. In neonates, caspofungin is the only echinocandin indicated for salvage therapy for refractory invasive candidiasis. Anidulafungin is not routinely recommended in children due to lack of pharmacokinetic studies in children. Echinocandins have also been used as first-line agents for infections by the multidrug-resistant *Candida auris* [32]. Echinocandins have no activity against cryptococcosis. The clinical uses and indications for echinocandins are shown in Table 5 [13, 33].

## 7. Conclusions

With the increase in prevalence of IFI, antifungal agents are increasingly needed in the pediatric critical care setting. Each class has its advantages and first-line indications. Knowledge of spectrum of activity and indications of antifungals are imperative for prompt treatment of critically ill children with IFI.

## Figures and Tables

**Table 1:** United States Food and Drug Administration (USFDA) approval timeline of antifungal agents [13–15].

Year of approval	Name of the antifungal agent
1958	Amphotericin B deoxycholate
1973	5-Flucytosine
1981	Ketoconazole
1990	Fluconazole
1992	Itraconazole (oral suspension in 1997; intravenous form in 1999)
1995	Amphotericin B lipid complex
1996	Amphotericin B colloidal dispersion
1997	Liposomal amphotericin B
2001	Caspofungin
2002	Voriconazole
2005	Micafungin
2006	Anidulafungin
2006	Posaconazole (delayed release of tablet in 2013; intravenous form in 2014)
2015	Isavuconazole (Isavuconazonium) (oral and intravenous)

**Table 2:** Spectrum of activity of antifungals against fungi causing invasive disease in critical care [13, 14].

Antifungal agent	*Aspergillus*	*Candida*	*Cryptococcus*	*Zygomyces* (*Mucor*, *Rhizopus*)	*Fusarium*	*Scedosporium*
Amphotericin B	+	+	+	+	−	−
Fluconazole	−	+	+	−	−	−
Itraconazole	+	+	+	−	+/−	−
Voriconazole	+	+	+	−	+	+
Posaconazole	+	+	+	+	+	+
Caspofungin	+	+	−	−	−	−
Micafungin	+	+	−	−	−	−
Anidulafungin	+	+	−	−	−	−

**Table 3:** Clinical indications and pediatric dosing of conventional and liposomal preparations of amphotericin B [13, 21].

Drug	Indications	Dose	Special comments
Amphotericin B	Neonates with disseminated candidiasis including CNS disease candidiasis, invasive candidiasis, mucosal aspergillosis cryptococcal meningitis coccidioidomycosis histoplasmosis blastomycosis mucormycosis sporotrichosis	1 mg/kg/day intravenously	Conventional preferred for 1. Neonatal candidiasis 2. Chorioretinitis with vitritis (intravitreal injection of 5–10 *µ*g/0.1 mL sterile water) 3. Intraventricular use: device-related CNS candidiasis; when device cannot be removed: amphotericin B intraventricular through device at 0.01 mg–0.5 mg in 2 mL 5% dextrose in water

Liposomal amphotericin B	Same as above with exceptions noted above	3–5 mg/kg/day intravenously	

**Table 4:** Clinical indications and pediatric dosing of azoles [13, 22].

Drug	Indications	Dose	Special comments
Fluconazole	Candidiasis, invasive candidiasis, mucosal cryptococcosis *Candida* prophylaxis in neonates	12 mg/kg loading dose, then 6 mg/kg QD	Empiric therapy for suspected candidiasis in nonneutropenic patients —fluconazole-resistant species of *Candida* are *C. krusei* and *C. glabrata* —all isolates of *Candida* should have susceptibility testing for azoles

Itraconazole	Candidiasis Coccidioidomycosis Histoplasmosis Blastomycosis Sporotrichosis Paracoccidioidomycosis	2.5–5 mg/kg twice-thrice per day	It is a second-line agent for aspergillosis

Voriconazole	Aspergillosis Candedemia in febrile neutropenia Invasive candidiasis, Fusariosis Scedosporiosis	4–7 mg/kg every 12 h	Voriconazole is the preferred agent for aspergillosis but has no activity against zygomycosis

**Table 5:** Clinical indications and dosing of echinocandins [13, 33].

Drug	Indications	Dose	Special comments
Caspofungin	Candidemia in neutropenic and nonneutropenic patients Empiric therapy for suspected candidiasis in nonneutropenic patients with risk factors for invasive candidiasis *Candida* osteoarticular infections Second-line therapy for aspergillosis	Caspofungin: 50 mg/m2/d and 25 mg/m2/day in neonates and infants <3 months	First-line for empiric therapy of candidiasis in patients with febrile neutropenia Consider susceptibility testing in patients with prior treatment and with *Candida glabrata* or *Candida parapsilosis* (may be resistant)

Micafungin	Same as above	1–3 mg/kg/d	

Anidulafungin	Mucosal or invasive candidiasis	Age >16 years: 100–200 mg for 1 dose, then 50–100 mg/d	Not approved for pediatric use

## Abbreviations

IFI: Invasive fungal infections
ICU: Intensive care unit.
